# The effect of CNQX on self-administration: present in nicotine, absent in methamphetamine model

**DOI:** 10.3389/fnbeh.2023.1305412

**Published:** 2024-01-05

**Authors:** Maria Hrickova, Petra Amchova, Jana Ruda-Kucerova

**Affiliations:** Department of Pharmacology, Faculty of Medicine, Masaryk University, Brno, Czechia

**Keywords:** AMPA/kainate receptor, CNQX, nicotine, methamphetamine, self-administration, relapse

## Abstract

**Objective:**

Addiction is a chronic disease with limited pharmacological options for intervention. Focusing on reducing glutamate levels in the brain seems to be a promising strategy in addiction treatment research. Our research aimed to evaluate the effects of CNQX, an antagonist that targets AMPA and kainate glutamatergic receptors while also exhibiting affinity for the NMDA receptor, especially by modulating its glycine site. We conducted this assessment on the self-administration of nicotine and methamphetamine via intravenous (IV) administration in rats.

**Methods:**

An operant IV self-administration model was used in male Wistar rats. When animals maintained a stable intake of nicotine or methamphetamine, we administered a single injection of CNQX (in the dose of 3 or 6 mg/kg IV) to evaluate its effect on drug intake. Subsequently, the rats were forced to abstain by staying in their home cages for 2 weeks. The period of abstinence was followed by a context-induced relapse-like session before which animals were pretreated with the injection of CNQX (3 or 6 mg/kg IV) to evaluate its effect on drug seeking.

**Results:**

CNQX significantly reduced nicotine intake during the maintenance phase, but no effect was revealed on nicotine seeking after forced abstinence. CNQX did not affect methamphetamine taking or seeking.

**Conclusion:**

The effect of reducing nicotine taking but not seeking could be explained by different involvement of glutamatergic receptors in various stages of nicotine dependence.

## 1 Introduction

Abused substances feature different origins, legal status, abuse liability, exert different pharmacological effect, harm on the user and are responsible for different socioeconomic burden. One of the drugs usually accepted by society is nicotine - an active ingredient of tobacco leaves. Nicotine acts mainly on nicotinic acetylcholine receptors; increasing alertness, memory function, blood pressure and heart rate (Prochaska and Benowitz, 2019). The most common way to consume tobacco is through inhalation via cigarettes, but recently vaping devices, nicotine pouches and lozenges are gaining popularity due to heavy advertising and the mistaken belief that they provide a healthier way to consume nicotine (Dinardo and Rome, 2019; Jenssen and Wilson, 2019; Walley et al., 2019; Unger et al., 2022). Given that nicotine is readily available to the general public (Cwalina et al., 2021), its addictive properties pose a significant health hazard that may continue to grow in the future (Dinardo and Rome, 2019). Although there are some approved pharmacotherapies for nicotine addiction (Tobacco Use and Dependence Guideline Panel, 2008), they have limitations (Polosa and Benowitz, 2011). Thus, searching for new pharmacotherapies for nicotine addiction is still essential.

In contrast, there is methamphetamine, a synthetic substance that is widely deemed illegal (Courtney and Ray, 2014). It is highly addictive due to its ability to target multiple neural pathways, all of which lead to a robust increase of monoamines in the reward pathway. Methamphetamine is typically administered intravenously, orally, sniffed or smoked, resulting in euphoric stimulant effects, anorexia, and sleep deprivation (Cruickshank and Dyer, 2009). Methamphetamine addiction, despite its extensive history (Grobler et al., 2011) and its severe impact on both physical and mental health (Darke et al., 2008), continues to rise worldwide (Han et al., 2021; Lewis et al., 2021). Importantly, there is no approved pharmacological treatment (Chan et al., 2019), which makes it a challenging issue to research and resolve.

This study focuses on both drugs – nicotine and methamphetamine – with very different pharmacodynamics (Tiwari et al., 2020; Yasaei and Saadabadi, 2022). Despite their differences, both substances eventually lead to increase dopamine release in the reward pathway (Volkow et al., 2011), as well as increased glutamatergic signaling in the cortex, nucleus accumbens, hippocampus and striatum (Gass and Olive, 2008). Although accumbal dopaminergic mechanisms are necessary for reinforcing effects of drugs, their chronic use leads to predominantly cortical neuroadaptations mediated by glutamate that contribute to the development of addiction in human (Volkow et al., 2019; Picciotto and Kenny, 2021).

The glutamate homeostasis hypothesis connects addiction to an imbalance in glutamate levels, causing alterations in brain circuits and diminishing control over drug-seeking behavior, thereby increasing vulnerability to relapse (Kalivas, 2009; Fischer et al., 2021). Notably, elevated glutamate release in the nucleus accumbens is a prominent feature in the reinstatement of extinguished drug-seeking, observed across various substances, including nicotine and methamphetamine (Scofield et al., 2016). The underlying neurotransmission changes involve differential subunit composition of the glutamatergic receptors (Gass and Olive, 2008; Stone, 2021), colocalization of ionotropic glutamatergic receptors with other receptor types (Volkow et al., 2019) and involvement of glial cells and astrocytes in the neuroadaptations following substance use (Fouyssac and Belin, 2019).

There seems to be a particular yet inconclusively described role of α-amino-3-hydroxy-5-methyl-4-isoxazolepropionic acid (AMPA) receptors in the human substance abusers pointing to a single relatively consistent outcome – an increase in the hippocampal expression of AMPA receptor subunits (Ueno et al., 2019). In preclinical research, multiple drugs were shown to exert alterations of AMPA receptor subunits’ expression in reward-related brain areas, typically nucleus accumbens or ventral tegmental area, e.g., cocaine (Sutton et al., 2003), amphetamine (Cruz et al., 2008), nicotine (Wang et al., 2007) or methamphetamine (Scheyer et al., 2016; Murray et al., 2019). Hence, the potential of pharmacological manipulation of AMPA receptors has been repeatedly suggested as a treatment target for SUD (Lee et al., 2016; Cooper et al., 2017; Ueno et al., 2019). Specifically, it has been shown that injecting AMPA receptors agonist into NAc decreased cocaine intake – suggesting that glutamate transmission augments the threshold of the reinforcing effect of cocaine intake (Cornish et al., 1999). On the other hand, microinjections of AMPA agonist reinstated cocaine-seeking behavior (Cornish et al., 1999; Cornish and Kalivas, 2000; Kruzich and Xi, 2006), and conversely, intra-NAc injections of AMPA antagonists were able to attenuate it (Cornish and Kalivas, 2000; Di Ciano and Everitt, 2001; Park et al., 2002; Ciano and Everitt, 2004; Bäckström and Hyytiä, 2007). Moreover, during the withdrawal of the drugs, expression of AMPA receptors tends to be increased in NAc (Boudreau, 2005; Ferrario et al., 2011; Moretti et al., 2020), which offers a possible explanation for the known phenomenon of drug craving and relapse (Cornish and Kalivas, 2000).

However, there is currently limited evidence supporting the use of AMPA/kainate antagonists as a potential treatment for nicotine and methamphetamine dependence behavior. Therefore, in this study, we used an AMPA/kainate antagonist, CNQX [which also has an affinity for the NMDA receptor’s glycine site (Davies, 2007)], to evaluate its effect on the operant drug-taking and drug-seeking in two pharmacologically distinct model drugs: nicotine and methamphetamine.

## 2 Materials and methods

### 2.1 Animals

Fifty male albino Wistar rats (8–9 weeks old) were purchased from the Masaryk University breeding facility (Brno, Czech Republic). Rats were initially housed in pairs in standard plastic rodent cages. They were housed individually after surgery and for the rest of the study. Forty rats were included in the self-administration studies (20 for nicotine and 20 for methamphetamine study). Some animals were lost due to general anesthesia during surgery, and the rest was removed based on catheter patency.

Environmental conditions during the study were: relative humidity 50–60%, room temperature 22°C ± 1°C, and inverted 12-h light–dark cycle (6 a.m. to 6 p.m. darkness). Water was available *ad libitum* throughout the study; food intake was restricted to 20 g/day except for the recovery period. Also, rats were food deprived for 24 h before training with sucrose pellets. All procedures were performed following EU Directive no. 2010/63/EU and approved by the Animal Care Committee of the Faculty of Medicine, Masaryk University, Czech Republic and Czech Governmental Animal Care Committee, in compliance with Czech Animal Protection Act No. 246/1992.

### 2.2 Drugs and treatments

Nicotine was purchased from Alomone Labs Ltd. (Jerusalem, Israel) as (−)-nicotine ditartrate and dissolved in saline to obtain a dose of 0.03 mg/kg in 0.1 mL (calculated as a free base). This dose is routinely used in nicotine self-administration studies (Fattore et al., 2009; Pushparaj et al., 2015; Boutros et al., 2016; Ruda-Kucerova et al., 2021). The maximum number of infusions in one session was not limited.

Methamphetamine (METH) was purchased from Sigma Chemical, Co., St Louis, MO, United States; IV self-administration was 0.08 mg/kg per infusion with the maximum number of infusions in one session set at 60 as previously described and validated (Amchova et al., 2014; Ruda-Kucerova et al., 2015a, 2017, 2021; Babinska et al., 2016). This approach prevents potentially dangerous overdose, which may happen with strong reinforcers such as METH.

**Figure 1:**
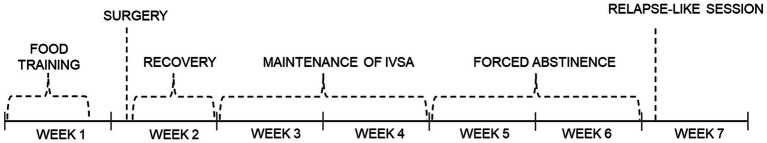
Schematic timeline of both nicotine and methamphetamine study.

CNQX was purchased from Alomone Labs Ltd. (Jerusalem, Israel) and dissolved in saline to obtain a 2 mg/mL concentration. CNQX was administered intravenously (IV) to the intrajugular catheter in the volume of 1.5 mL/kg for the dose of 3 mg/kg and 3 mL/kg for the dose of 6 mg/kg 10 minutes before the operant session. Saline was used as vehicle. The doses and administration time were selected based on already existing studies (Bäckström and Hyytiä, 2006; Wooters et al., 2011) and our previous CNQX pilot study. While CNQX is typically administered intraperitoneally, we opted for intravenous administration due to the presence of intravenous catheters in rats. This approach was chosen to minimize invasiveness, refine the procedure, to prevent potential pre-systemic elimination and to achieve 100% bioavailability. Each rat received acute doses of SAL, CNQX 3 or 6 mg/kg, or a subset of these treatments, following Latin square design. The administration began after at least 8 days of operant drug self-administration, when a stable intake was reached. Stable intake was defined in terms of mean number of injections ±25%. In the last session, after abstinence animals received SAL, CNQX 3 or 6 mg pseudorandomly, ensuring that rats with the same history of CNQX/vehicle exposure were included in all test groups. The timeline of the operant studies is shown in Figure 1.

### 2.3 Food self-administration protocol

Food self-administration was used to train operant behavior in the animals. The training was conducted as previously described (Ruda-Kucerova et al., 2015a, 2017, 2021; Babinska and Ruda-Kucerova, 2017). Briefly, ten operant boxes (30x25x30 cm, Coulbourn Instruments, United States) provided with two nose-poke holes allocated on one side and programmed by software Graphic State Notation 3.03 (Coulbourn Instruments, United States) were used under a fixed ratio 1 (FR-1) schedule of reinforcement to obtain a single palatable pellet (BioServ, sweet dustless rodent pellets, F0021-Purified Casein Based Formula - 45 mg, sweet taste attributed by 276 g/kg of monosaccharides, and 310 g/kg of sucrose). Throughout the entire 30-min session, a house light provided illumination inside the cage. This session occurred during the dark phase of a reversed light–dark cycle and lasted for five consecutive days. No discrete cues were included during this period. Food self-administration training preceded both nicotine as well as methamphetamine study.

### 2.4 IV drug self-administration (IVSA) protocol

Animals were deeply anesthetized with isoflurane inhalation. Under aseptic conditions, a permanent intracardiac catheter was implanted through the external jugular vein to the right atrium. The outer part of the catheter exited the skin in the midscapular area. After surgery, a one-week recovery was allowed. The catheters were flushed daily with enrofloxacin (17 mg/kg) solution followed by 0.1 mL of a heparinized (1%) sterile saline solution to prevent infection and occlusion of the catheter. IVSA was conducted as previously described (Amchova et al., 2014; Ruda-Kucerova et al., 2015a, 2017; Babinska et al., 2016) in the same operant boxes (Coulbourn Instruments, United States) using nose-poke operanda under a FR-1. When the infusion was accessible, the active nose poke became illuminated. Any stimulation in the active nose-poke led to administration of the infusion. During the infusion (8 s), the nose-poke illumination went off and the house light was flashing, providing environmental cue linked to the drug infusion. Following this, a 20-s time-out was enforced, during which nose-poking was recorded but was not associated with any rewards, the house light and the cue light were off. Nicotine IVSA sessions lasted 60 min, and METH IVSA sessions lasted 90 min. IVSA was performed 7 days/a week between 9 a.m. and 1 p.m. during the dark period of the inverted light–dark cycle, after which the rats were returned to their home cages. After 2 weeks of drug intake, the maintenance phase was terminated, whereby the rats were kept in their home cages for another 14 days of forced abstinence. On the 15th day of abstinence, rats were once more introduced to IVSA chambers for a drug-free relapse-like session. The purpose of this session was to induce drug-seeking behavior based on the specific environment associated with drug intake. The cue lights were off and house light on throughout the entire session. The cannulas were connected to the animals, but no solution was delivered.

### 2.5 Statistical data analysis

Primary data were summarized using arithmetic mean and standard error of the mean (±SEM) estimate. The treatment effect was analyzed by one-way ANOVA followed by Tukey post-hoc test when appropriate. The analyses were calculated using Statistica 13.5.0.17 (Tibco Software Inc., United States). A value of *p* < 0.05 was recognized as the boundary of statistical significance in all applied tests.

## 3 Results

**Figure 2:**
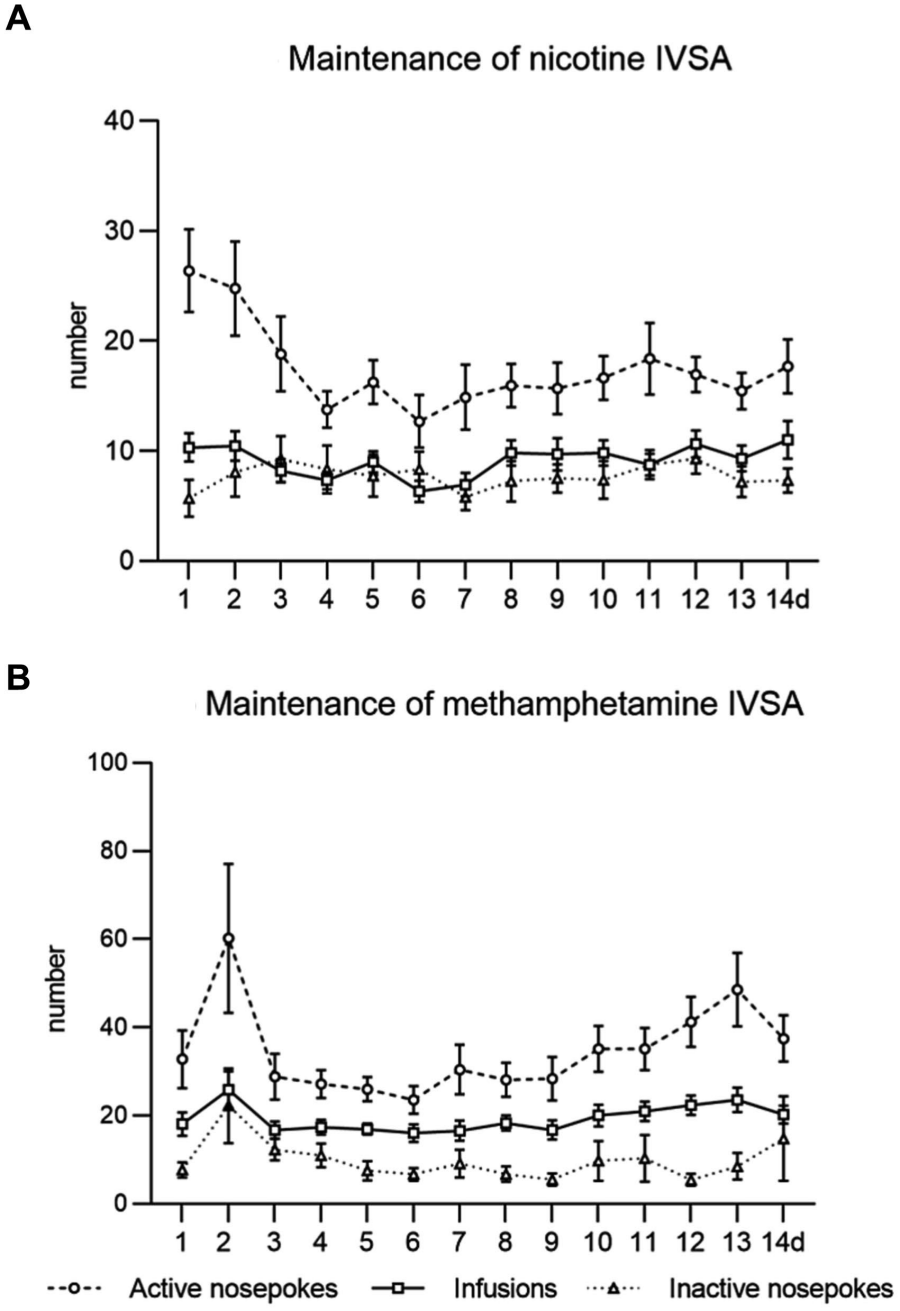
Maintenance of intravenous self-administration of nicotine **(A)** and methamphetamine **(B)**. The line graphs show mean values ± SEM of active nose-pokes, inactive nose-pokes, and the number of infusions obtained daily during the IVSA maintenance period.

In both studies, we observed the expected acquisition of drug intake, stable responses and drug self-administration over 2 weeks of the maintenance period. Data from the maintenance training of both studies are shown in Figure 2.

Followed parameters during maintenance sessions were responding to the drug by active nose-pokes, number of self-administered infusions, and drug intake in mg/kg. In the context-induced relapse-like session, we analyzed responses for the drug by active nose-pokes, inactive nose-pokes and active operandum preference (% of previously drug-paired responses over the total number of responses).

### 3.1 Nicotine study

**Figure 3:**
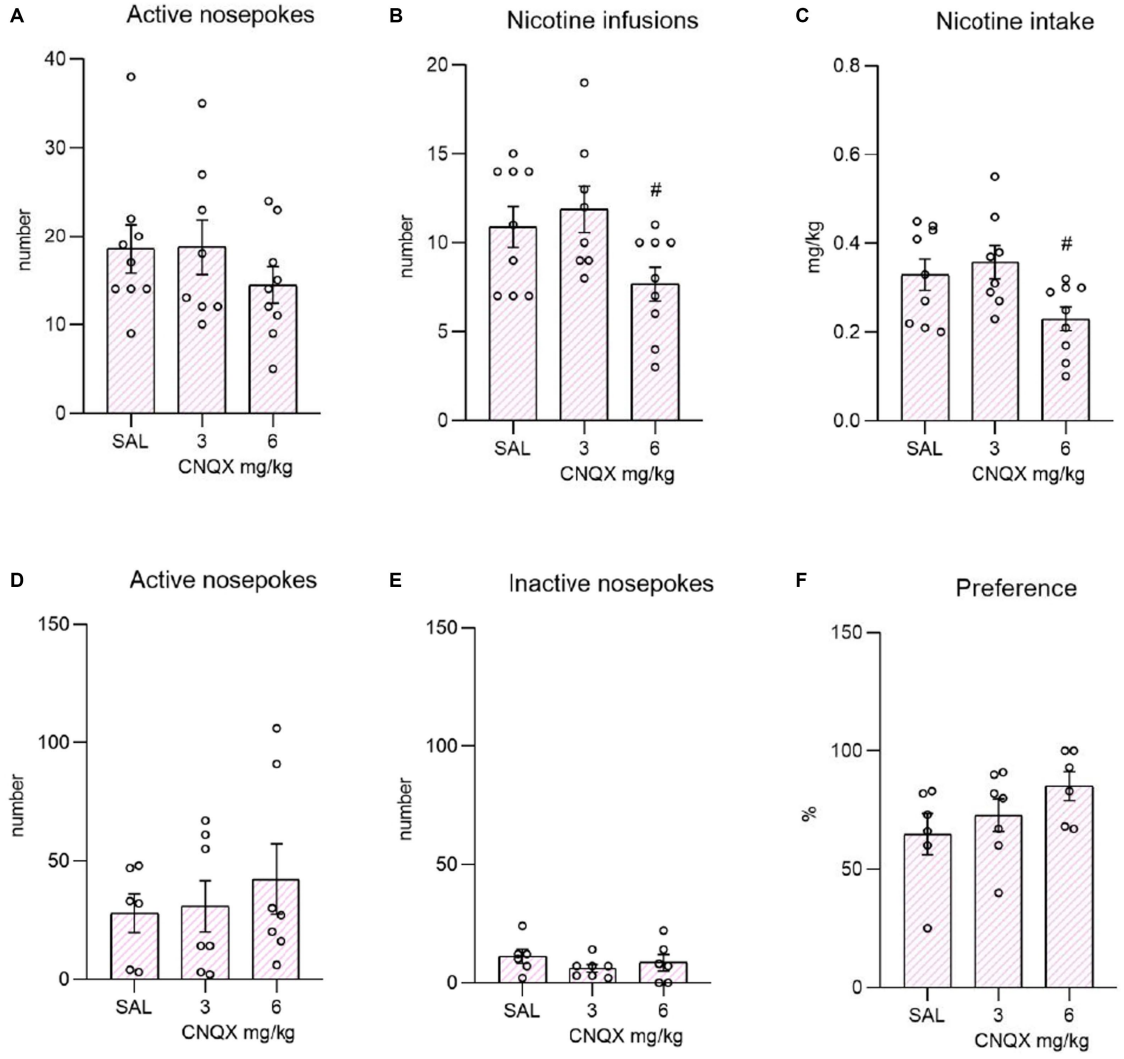
Depicts the effect of CNQX treatment in the nicotine study. The bar graphs on the top of the panel show mean values ± SEM of nicotine operant variables after CNQX treatment. Graphs indicate the effect of CNQX treatment at doses of 3 and 6 mg/kg during maintenance on active nose-pokes **(A)**, the number of infusions **(B)**, and nicotine dose in mg/kg **(C)**. ^#^(*p* = 0.042) indicates a significant difference between CNQX3 and CNQX6 groups in nicotine infusions **(B)** and ^#^(*p* = 0.035) nicotine dose in mg/kg **(C)**. Graphs at the bottom indicate the effect of CNQX treatment at doses of 3 and 6 mg/kg during the context-induced nicotine seeking after forced abstinence on active nose-pokes **(D)**, inactive nose-pokes **(E)**, and preference of the active operandum **(F)** - % of previously drug-paired responses over the total number of responses.

The data from the nicotine study are depicted in the Figure 3. The top bar graphs present nicotine operant variables post-CNQX treatment. The lower graphs portray the effects of CNQX treatment on the nicotine seeking after forced abstinence. Data were analyzed by one way-ANOVA which revealed no differences in active (F_2,23_ = 0.850, *p* = 0.440) or inactive (F_2,23_ = 2.070, *p* = 0.149) responding, while CNQX treatment significantly decreased the number of self-administered injections (F_2,23_ = 3.751, *p* = 0.039), as well as the nicotine intake (F_2,23_ = 4.034, *p* = 0.031). Tukey post-hoc test indicated a significant effect of the higher CNQX dose compared to the lower dose in both variables, specifically: the number of infusions, *p* = 0.042 and nicotine dose, *p* = 0.035. The post-hoc test did not show the difference between vehicle control and the 6 mg/kg dose.

After the forced abstinence, in the nicotine-free relapse-like session, one-way ANOVA did not reveal a significant effect of CNQX treatment on the number of active nose-pokes (F_2,17_ = 0.406, *p* = 0.672833) or inactive nose-pokes (F_2,16_ = 0.895, *p* = 0.428). The percent of preference for the active operandum was equally high among the groups (F_2,16_ = 1.844, *p* = 0.190).

### 3.2 Methamphetamine study

**Figure 4:**
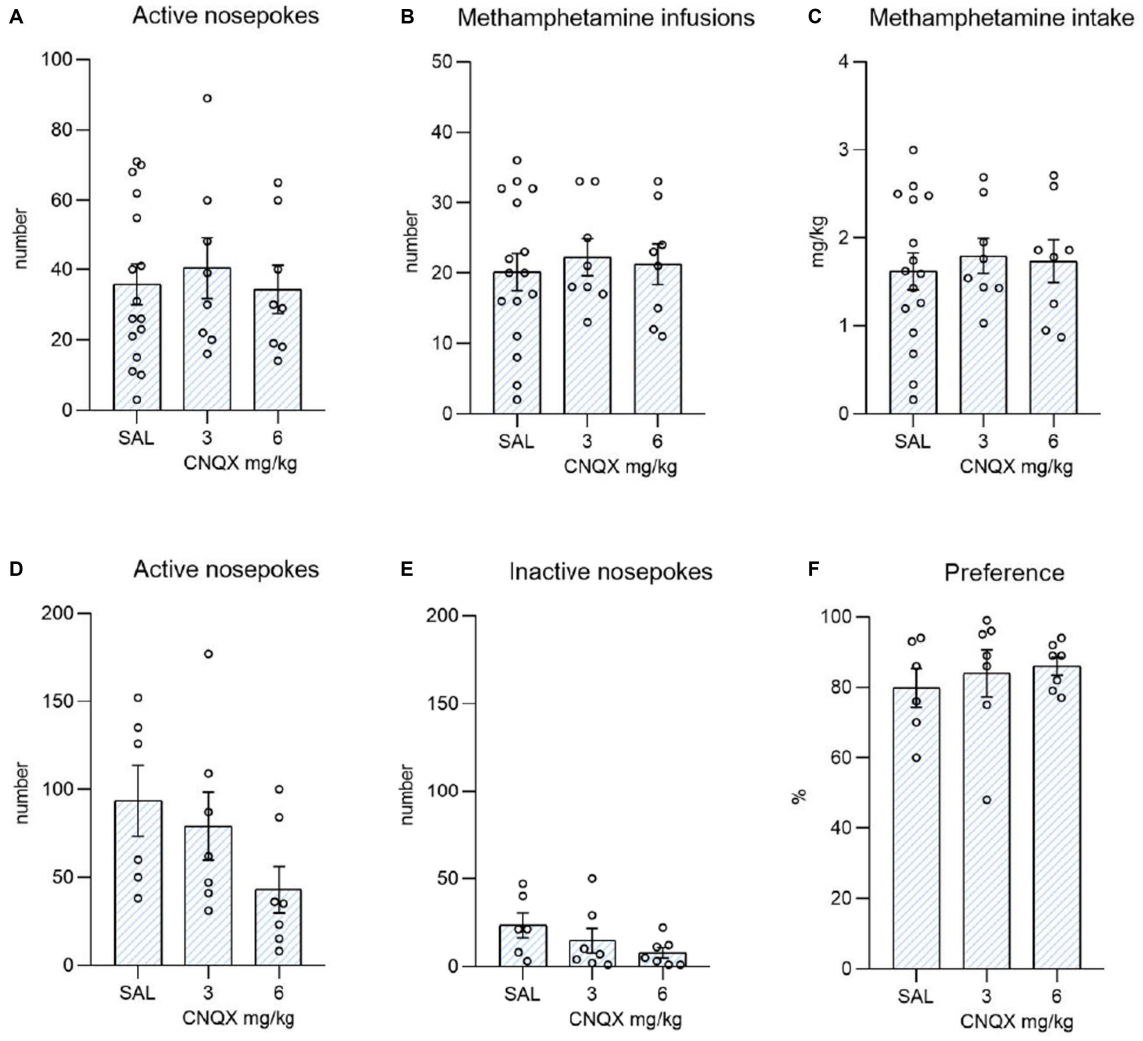
Depicts the effect of CNQX treatment in the METH study. The bar graphs show mean values ± SEM of methamphetamine operant variables after CNQX treatment. Graphs at the top indicate the effect of CNQX treatment at doses of 3 and 6 mg/kg during maintenance on active nose-pokes **(A)**, the number of infusions **(B)**, and METH dose in mg/kg **(C)**. Graphs at the bottom indicate the effect of CNQX treatment at doses of 3 and 6 mg/kg during the context-induced methamphetamine seeking after forced abstinence on active nose-pokes **(D)**, inactive nose-pokes **(E)**, and preference of the active operandum **(F)** - % of previously drug-paired responses over the total number of responses. No significant differences were revealed.

The data from the methamphetamine study are shown in Figure 4. The top graphs depict the influence of CNQX treatment during the maintenance phase of METH self-administration. The bottom graphs convey the effect of CNQX treatment during context-induced methamphetamine seeking after forced abstinence. Data were analyzed in the same manner as in the nicotine study. One-way ANOVA did not reveal any effect of CNQX treatment either on active (F_2,29_ = 0.168, *p* = 0.846) or inactive (F_2,29_ = 1,161, *p* = 0,327) responding, nor on self-administered injections (F_2,29_ = 0.144, *p* = 0.867) or dose of methamphetamine intake (F_2,29_ = 0.168, *p* = 0.847).

In the final session, after forced abstinence, no difference was found in the active operandum’s preference (F_2,17_ = 0.348, *p* = 0.711), as well as in the number of active nose-pokes (F_2,17_ = 2.160, *p* = 0.146), and the number of inactive nose-pokes (F_2,17_ = 1.711, *p* = 0.210).

## 4 Discussion

The findings from this research show a decrease in nicotine consumption after a single administration of a higher dose (6 mg/kg) of CNQX. However, CNQX did not have any impact on drug-seeking behavior during a relapse-like session following the forced abstinence. In the methamphetamine study, no effect of CNQX was observed in any phase of the operant protocol.

Quinoxalinediones, such as CNQX, are a group of ligands that are specifically designed to target AMPA receptors. However, many AMPA receptor antagonists also block kainate receptors, making the molecular targets broader and attenuating glutamate in a more complex manner (Gass and Olive, 2008). CNQX, for example, is a competitive antagonist on AMPA/kainate receptors, but it also has an affinity towards the glycine site of NMDA receptors (Lester et al., 1989), which may contribute to its effect on drug-related behaviors. Studies have shown that NMDA/glycine site antagonists alone can reduce cue-induced cocaine reinstatement (Bäckström and Hyytiä, 2006), prevent the expression of amphetamine-induced conditioned place preference (Mead and Stephens, 1999), and decrease ethanol-seeking behavior (Bäckström and Hyytiä, 2004).

Only a few behavioral studies have explored the impact of AMPA/kainate antagonists on the nicotine dependence model, with even fewer dedicated specifically to the self-administration. The results of these studies indicate that MPQX, a competitive AMPA antagonist, can decrease nicotine-induced dopamine release in the nucleus accumbens core and reduce the increase in locomotor activity caused by nicotine. However, NBQX, a substance that also has an affinity to kainate receptors, did not impact either of these processes (Kosowski et al., 2004). This is consistent with previous studies showing that NBQX had no effect on nicotine intake (Kenny et al., 2009; Ruda-Kucerova et al., 2021). On the other hand, antagonism on NMDA receptors blocked nicotine-induced lowering of intracranial self-stimulation thresholds, attenuated nicotine self-administration (Kenny et al., 2009), decreased nicotine-induced dopamine release and locomotor activity (Kosowski et al., 2004). These findings and our study suggest that the mechanism of CNQX responsible for reduction of nicotine intake is rather NMDA than AMPA or kainate receptors antagonism.

Conversely, we demonstrated no effect of CNQX on nicotine-seeking after two weeks of forced abstinence, while in our previous study, NBQX was able to reduce it (Ruda-Kucerova et al., 2021). This may indicate that AMPA/kainate receptors are more involved in the post-abstinence relapse-like phase of nicotine seeking behavior. However, currently there is no more studies testing quinoxalinediones in models of nicotine dependence to support this notion.

In the model of operant methamphetamine self-administration, we observed no impact of CNQX on methamphetamine maintenance or its seeking in the relapse-like session. Behavioral research of AMPA/kainate receptors and dependence to stimulants includes a number of studies with cocaine, where both CNQX and NBQX were able to attenuate drug seeking (Bäckström and Hyytiä, 2003, 2006; Zavala et al., 2008). However, NBQX showed a difference in its effect depending on how the abstinence was induced: the effect was shown only in the group in which abstinence was forced, not when animals underwent extinction protocol (Zavala et al., 2008). What seems more consistent are data from cocaine studies where AMPA/kainate antagonists were injected directly into a specific part of the brain, resulting in a higher concentration and increased bioavailability at the site of action. E.g., when these ligands were infused into the nucleus accumbens core, cocaine intake was decreased (Suto et al., 2009) as well as cocaine-seeking behavior induced by a variety of factors (Cornish and Kalivas, 2000; Di Ciano and Everitt, 2001; Park et al., 2002; Ciano and Everitt, 2004; Bäckström and Hyytiä, 2007; Xie et al., 2012). The effect was absent when infused into the nucleus accumbens shell (Di Ciano and Everitt, 2001; Suto et al., 2009). Based on these studies, AMPA/kainate antagonists may play a role in cocaine-seeking behavior through their effects on the nucleus accumbens core. However, there is limited knowledge regarding the influence of AMPA/kainate antagonists on behavior associated with other stimulant drugs, particularly methamphetamine, and results are overall contradictory. For instance, NBQX did not attenuate behavioral sensitivity induced by amphetamine (Li et al., 1997) but did attenuate amphetamine (Dalia and Wallace, 1995; Mead and Stephens, 1998; Vanover, 1998) and methamphetamine-induced locomotor activity (Witkin, 1993). Yet in another study, several days of pretreatment with NBQX did not attenuate methamphetamine-induced hyperactivity (Akiyama et al., 1998).

In amphetamine-conditioned place preference protocol, CNQX or DNQX was able to block its acquisition (Layer et al., 1993; Mead and Stephens, 1999) and also its expression (Miyatake et al., 2005; Banasikowski et al., 2012). In stark contrast, NBQX was ineffective, did not block the amphetamine-conditioned place preference (Mead and Stephens, 1998, 1999) and caused conditioned place aversion at a 30 mg/kg dose (Mead and Stephens, 1999). Moreover, in operant self-administration studies, NBQX did not reduce the intake of methamphetamine nor its seeking in relapse (Ruda-Kucerova et al., 2021). Similarly, CNQX did not affect the stimulant effect of methamphetamine on self-administration of palatable pellets (Wooters et al., 2011). Based on these findings, it seems unlikely that CNQX or AMPA/kainate antagonists in general, represent a promising treatment for methamphetamine dependence. However, the effect of administering antagonists directly to specific brain regions has not yet been studied in depth. Given the variations in the mechanisms of action among different stimulants, the effectiveness of AMPA/kainate antagonist treatment may differ depending on the specific drug. For example, when comparing the behavioral effects and subsequent neuroadaptations related to incubation of craving for cocaine and methamphetamine, numerous distinctions in glutamate synaptic plasticity in the nucleus accumbens core were reported (Scheyer et al., 2016; Murray et al., 2019; Funke et al., 2023). Hence, it is important to note that cocaine and amphetamine should not be grouped together when discussing the role of glutamate-related plasticity. Specifically, the results from studies on AMPA receptors for one drug cannot be applied to the other (Wolf and Ferrario, 2010).

The effectiveness of CNQX in reducing self-administration of nicotine but not methamphetamine may be due to the difference in dopamine release in the reward pathway caused by these drugs and the effect of glutamate on dopamine release. The amount of dopamine released after nicotine injection is significantly lower than the amount which is released after stimulants (Di Chiara and Imperato, 1988; Scherma et al., 2008; Ruda-Kucerova et al., 2015b). Since glutamate release modifies dopamine release (Tzschentke and Schmidt 2003), this difference in dopamine release may explain why a dose of 6 mg/kg of CNQX effectively reduced self-administration of nicotine but not methamphetamine. However, further increasing the dose of CNQX could negatively impact spontaneous locomotor activity, which was reported to be partially affected already by 3 mg/kg of CNQX (Bäckström and Hyytiä, 2003). Nevertheless, in our study, the 6 mg/kg dosage did not affect locomotor activity, as evidenced by the lack of a significant effect on both inactive and active nose pokes. Another aspect to consider is the different mechanisms of action between these two drugs. However, both of these drugs share the discriminative stimulus effect as shown in a model of operant food self-administration (Gatch et al., 2008). Hence, despite their pharmacological differences, they are likely to induce craving.

Notably, use of CNQX in animal research has some methodological limitations, such as its pH-sensitive binding to the AMPA receptor GluA2 LBD (Dudić and Reiner, 2019), poor solubility, and limited ability to penetrate the central nervous system (Bigge and Nikam, 1997), which can affect its bioavailability and dose-dependent efficacy. As of our present knowledge, a comprehensive pharmacokinetic investigation following different routes of CNQX administration is lacking. Nevertheless, the route of administration can significantly impact effects of any drug. The prevailing method of CNQX administration predominantly employs intraperitoneal administration. Our use of IV route can be expected to have earlier onset of effect, 100% bioavailability and higher peak plasma concentration. We reflect these pharmacokinetic aspects in the experimental design by administering CNQX only10 minutes before the self-administration session. Another aspect to consider is the behavioral protocol, as the impact of short-term versus extended drug exposure has been established. Research has shown that CNQX, when administered systemically or directly to the nucleus accumbens, only decreased cocaine seeking in the group with prolonged drug exposure (Doyle et al., 2014; Lynch et al., 2021). Hence, further research is needed to investigate the impact of CNQX in studies allowing extended drug access. Additionally, it would be interesting to explore the potential of CNQX in altering the affective state. There is evidence of CNQX affecting the emotional aspect of pain (Spuz and Borszcz, 2012; Yan et al., 2012). Tracking this effect could be particularly interesting in the context of craving, as reported as the case of nicotine-degrading enzyme which normalized hyperalgesic reaction following nicotine withdrawal from extended access self-administration protocol (Kallupi et al., 2018).

Taken together, in this study, we found that the use of CNQX at a dose of 6 mg/kg significantly reduced nicotine response during the maintenance phase but not nicotine-seeking in the relapse-like session. We previously showed that NBQX reduces nicotine seeking in the relapse-like session but not nicotine intake (Ruda-Kucerova et al., 2021). This suggests a different role for glutamate receptors in nicotine dependence, as CNQX is not selective for AMPA or kainate receptors alone. Additionally, no effect of CNQX was observed on either methamphetamine intake or its seeking. We propose that the effects of CNQX on influencing drug-related behavior are drug-specific.

## Data availability statement

The raw data supporting the conclusions of this article will be made available by the authors, without undue reservation.

## Ethics statement

The animal study was approved by Expert committee for ensuring the welfare of experimental animals of the Faculty of Medicine, Masaryk University. The study was conducted in accordance with the local legislation and institutional requirements.

## Author contributions

MH: Conceptualization, Data curation, Investigation, Methodology, Visualization, Writing – original draft, Formal analysis. PA: Data curation, Writing – review & editing, Methodology. JR-K: Conceptualization, Funding acquisition, Investigation, Methodology, Project administration, Resources, Supervision, Writing – review & editing.

## References

[ref1] Akiyama K. Ujike H. Sakai K. Shimizu Y. Kodama M. Kuroda S. (1998). Effect of 2,3-dihydroxy-6-nitro-7-sulfamoyl-benzo(f)quinoxaline on methamphetamine- and cocaine-induced behavioral sensitization. Pharmacol. Biochem. Behav. 61, 419–426. doi: 10.1016/s0091-3057(98)00121-x 9802837

[ref2] Amchova P. Kucerova J. Giugliano V. Babinska Z. Zanda M. T. Scherma M.. (2014). Enhanced self-administration of the CB1 receptor agonist WIN55, 212-2 in olfactory bulbectomized rats: evaluation of possible serotonergic and dopaminergic underlying mechanisms. Front. Pharmacol. 5:44. doi: 10.3389/fphar.2014.00044 24688470 PMC3960502

[ref3] Babinska Z. Ruda-Kucerova J. (2017). Differential characteristics of ketamine self-administration in the olfactory bulbectomy model of depression in male rats. Exp. Clin. Psychopharmacol. 25:84. doi: 10.1037/pha0000106 28301174

[ref4] Babinska Z. Ruda-Kucerova J. Amchova P. Merhautova J. Dusek L. Sulcova A. (2016). Olfactory bulbectomy increases reinstatement of methamphetamine seeking after a forced abstinence in rats. Behav. Brain Res. SreeTestContent1 297, 20–27. doi: 10.1016/j.bbr.2015.09.035 26431766

[ref5] Bäckström P. Hyytiä P. (2003). Attenuation of cocaine-seeking behaviour by the AMPA/kainate receptor antagonist CNQX in rats. Psychopharmacology 166, 69–76. doi: 10.1007/s00213-002-1312-y 12525959

[ref6] Bäckström P. Hyytiä P. (2004). Ionotropic glutamate receptor antagonists modulate Cue-induced reinstatement of ethanol-seeking behavior. Alcohol. Clin. Exp. Res. 28, 558–565. doi: 10.1097/01.ALC.0000122101.13164.21 15100606

[ref7] Bäckström P. Hyytiä P. (2006). Ionotropic and metabotropic glutamate receptor antagonism attenuates Cue-induced cocaine seeking. Neuropsychopharmacology 31, 778–786. doi: 10.1038/sj.npp.1300845 16123768

[ref8] Bäckström P. Hyytiä P. (2007). Involvement of AMPA/kainate, NMDA, and mGlu5 receptors in the nucleus accumbens core in cue-induced reinstatement of cocaine seeking in rats. Psychopharmacology 192, 571–580. doi: 10.1007/s00213-007-0753-8 17347848

[ref9] Banasikowski T. J. MacLeod L. S. Beninger R. J. (2012). Comparison of nafadotride, CNQX, and haloperidol on acquisition versus expression of amphetamine-conditioned place preference in rats. Behav. Pharmacol. 23, 89–97. doi: 10.1097/FBP.0b013e32834ecb32 22157177

[ref10] Bigge C. F. Nikam S. S. (1997). AMPA receptor agonists, antagonists and modulators: their potential for clinical utility. Expert Opin. Ther. Pat. 7, 1099–1114. doi: 10.1517/13543776.7.10.1099

[ref11] Boudreau A. C. (2005). Behavioral sensitization to cocaine is associated with increased AMPA receptor surface expression in the nucleus Accumbens. J. Neurosci. 25, 9144–9151. doi: 10.1523/JNEUROSCI.2252-05.2005 16207873 PMC6725751

[ref12] Boutros N. Semenova S. Markou A. (2016). Adolescent alcohol exposure decreased sensitivity to nicotine in adult Wistar rats. Addict. Biol. 21, 826–834. doi: 10.1111/adb.12263 25950618 PMC4636981

[ref13] Chan B. Freeman M. Kondo K. Ayers C. Montgomery J. Paynter R.. (2019). Pharmacotherapy for methamphetamine/amphetamine use disorder—a systematic review and meta-analysis. Addiction 114, 2122–2136. doi: 10.1111/add.14755 31328345

[ref14] Ciano P. D. Everitt B. J. (2004). Direct interactions between the basolateral amygdala and nucleus Accumbens Core underlie cocaine-seeking behavior by rats. J. Neurosci. 24, 7167–7173. doi: 10.1523/JNEUROSCI.1581-04.2004 15306650 PMC6729170

[ref15] Cooper S. Robison A. J. Mazei-Robison M. S. (2017). Reward circuitry in addiction. Neurotherapeutics 14, 687–697. doi: 10.1007/s13311-017-0525-z 28324454 PMC5509624

[ref16] Cornish J. L. Duffy P. Kalivas P. W. (1999). A role for nucleus accumbens glutamate transmission in the relapse to cocaine-seeking behavior. Neuroscience 93, 1359–1367. doi: 10.1016/s0306-4522(99)00214-6 10501460

[ref17] Cornish J. L. Kalivas P. W. (2000). Glutamate transmission in the nucleus Accumbens mediates relapse in cocaine addiction. J. Neurosci. 20:RC89–RC89. doi: 10.1523/JNEUROSCI.20-15-j0006.2000 10899176 PMC6772531

[ref18] Courtney K. E. Ray L. A. (2014). Methamphetamine: an update on epidemiology, pharmacology, clinical phenomenology, and treatment literature. Drug Alcohol Depend. 143, 11–21. doi: 10.1016/j.drugalcdep.2014.08.003 25176528 PMC4164186

[ref19] Cruickshank C. C. Dyer K. R. (2009). A review of the clinical pharmacology of methamphetamine. Addiction 104, 1085–1099. doi: 10.1111/j.1360-0443.2009.02564.x 19426289

[ref20] Cruz F. C. Marin M. T. Planeta C. S. (2008). The reinstatement of amphetamine-induced place preference is long-lasting and related to decreased expression of AMPA receptors in the nucleus accumbens. Neuroscience 151, 313–319. doi: 10.1016/j.neuroscience.2007.10.019 18055123

[ref21] Cwalina S. N. McConnell R. Benowitz N. L. Barrington-Trimis J. L. (2021). Tobacco-free nicotine — new name, same scheme? N. Engl. J. Med. 385, 2406–2408. doi: 10.1056/NEJMp2111159 34919358 PMC9153389

[ref22] Dalia A. Wallace L. J. (1995). Amphetamine induction of c-fos in the nucleus accumbens is not inhibited by glutamate antagonists. Brain Res. 694, 299–307. doi: 10.1016/0006-8993(95)00794-Q 8974658

[ref23] Darke S. Darke S. Kaye S. Darke S. Kaye S. McKetin R.. (2008). Major physical and psychological harms of methamphetamine use. Drug Alcohol Rev. 27, 253–262. doi: 10.1080/09595230801923702 18368606

[ref24] Davies J. A. (2007). “CNQX” in xPharm: The comprehensive pharmacology reference. eds. Enna S. J. Bylund D. B. (New York: Elsevier), 1–3.

[ref25] Di Chiara G. Imperato A. (1988). Drugs abused by humans preferentially increase synaptic dopamine concentrations in the mesolimbic system of freely moving rats. Proc. Natl. Acad. Sci. U. S. A. 85, 5274–5278. doi: 10.1073/pnas.85.14.5274 2899326 PMC281732

[ref26] Di Ciano P. Everitt B. J. (2001). Dissociable effects of antagonism of NMDA and AMPA/KA receptors in the nucleus Accumbens Core and Shell on cocaine-seeking behavior. Neuropsychopharmacology 25, 341–360. doi: 10.1016/S0893-133X(01)00235-4 11522463

[ref27] Dinardo P. Rome E. S. (2019). Vaping: the new wave of nicotine addiction. Cleve. Clin. J. Med. 86, 789–798. doi: 10.3949/ccjm.86a.19118 31821136

[ref28] Doyle S. E. Ramôa C. Garber G. Newman J. Toor Z. Lynch W. J. (2014). A shift in the role of glutamatergic signaling in the nucleus Accumbens Core with the development of an addicted phenotype. Biol. Psychiatry 76, 810–815. doi: 10.1016/j.biopsych.2014.02.005 24629536 PMC4133320

[ref29] Dudić A. Reiner A. (2019). Quinoxalinedione deprotonation is important for glutamate receptor binding. Biol. Chem. 400, 927–938. doi: 10.1515/hsz-2018-0464 30903748

[ref30] Fattore L. Spano M. S. Cossu G. Scherma M. Fratta W. Fadda P. (2009). Baclofen prevents drug-induced reinstatement of extinguished nicotine-seeking behaviour and nicotine place preference in rodents. Eur. Neuropsychopharmacol. 19, 487–498. doi: 10.1016/j.euroneuro.2009.01.007 19250803

[ref31] Ferrario C. R. Li X. Wolf M. E. (2011). Effects of acute cocaine or dopamine receptor agonists on AMPA receptor distribution in the rat nucleus accumbens. Synapse 65, 54–63. doi: 10.1002/syn.20823 20506566 PMC2965794

[ref32] Fischer K. D. Knackstedt L. A. Rosenberg P. A. (2021). Glutamate homeostasis and dopamine signaling: implications for psychostimulant addiction behavior. Neurochem. Int. 144:104896. doi: 10.1016/j.neuint.2020.104896 33159978 PMC8489281

[ref33] Fouyssac M. Belin D. (2019). Beyond drug-induced alteration of glutamate homeostasis, astrocytes may contribute to dopamine-dependent intrastriatal functional shifts that underlie the development of drug addiction: a working hypothesis. Eur. J. Neurosci. 50, 3014–3027. doi: 10.1111/ejn.14416 30968489 PMC6852203

[ref34] Funke J. R. Hwang E.-K. Wunsch A. M. Baker R. Engeln K. A. Murray C. H.. (2023). Persistent neuroadaptations in the nucleus Accumbens Core accompany incubation of methamphetamine craving in male and female rats. eNeuro 10. doi: 10.1523/ENEURO.0480-22.2023 PMC10016192 36792361

[ref35] Gass J. T. Olive M. F. (2008). Glutamatergic substrates of drug addiction and alcoholism. Biochem. Pharmacol. 75, 218–265. doi: 10.1016/j.bcp.2007.06.039 17706608 PMC2239014

[ref36] Gatch M. B. Flores E. Forster M. J. (2008). Nicotine and methamphetamine share discriminative stimulus effects. Drug Alcohol Depend. 93, 63–71. doi: 10.1016/j.drugalcdep.2007.08.020 17961933 PMC2377183

[ref37] Grobler S. R. Chikte U. Westraat J. (2011). The pH levels of different methamphetamine drug samples on the street market in Cape Town. ISRN Dent 2011:974768. doi: 10.5402/2011/974768 21991491 PMC3189445

[ref38] Han B. Compton W. M. Jones C. M. Einstein E. B. Volkow N. D. (2021). Methamphetamine use, methamphetamine use disorder, and associated overdose deaths among US adults. JAMA Psychiatry 78, 1329–1342. doi: 10.1001/jamapsychiatry.2021.2588 34550301 PMC8459304

[ref39] Jenssen B. P. Wilson K. M. (2019). What is new in electronic-cigarettes research? Curr. Opin. Pediatr. 31:262. doi: 10.1097/MOP.0000000000000741 30762705 PMC6644064

[ref40] Kalivas P. W. (2009). The glutamate homeostasis hypothesis of addiction. Nat. Rev. Neurosci. 10, 561–572. doi: 10.1038/nrn2515 19571793

[ref42] Kallupi M. Xue S. Zhou B. Janda K. D. George O. (2018). An enzymatic approach reverses nicotine dependence, decreases compulsive-like intake, and prevents relapse. Sci. Adv. 4:eaat4751. doi: 10.1126/sciadv.aat4751 30345354 PMC6192681

[ref43] Kenny P. J. Chartoff E. Roberto M. Carlezon W. A. Markou A. (2009). NMDA receptors regulate nicotine-enhanced brain reward function and intravenous nicotine Self-administration: role of the ventral tegmental area and central nucleus of the amygdala. Neuropsychopharmacology 34, 266–281. doi: 10.1038/npp.2008.58 18418357 PMC2654386

[ref44] Kosowski A. R. Cebers G. Cebere A. Swanhagen A.-C. Liljequist S. (2004). Nicotine-induced dopamine release in the nucleus accumbens is inhibited by the novel AMPA antagonist ZK200775 and the NMDA antagonist CGP39551. Psychopharmacology 175, 114–123. doi: 10.1007/s00213-004-1797-7 15088078

[ref45] Kruzich P. J. Xi J. (2006). Different patterns of pharmacological reinstatement of cocaine-seeking behavior between Fischer 344 and Lewis rats. Psychopharmacology 187, 22–29. doi: 10.1007/s00213-005-0264-4 16418826

[ref46] Layer R. T. Uretsky N. J. Wallace L. J. (1993). Effects of the AMPA/kainate receptor antagonist DNQX in the nucleus accumbens on drug-induced conditioned place preference. Brain Res. 617, 267–273. doi: 10.1016/0006-8993(93)91094-9 8402155

[ref47] Lee K. Goodman L. Fourie C. Schenk S. Leitch B. Montgomery J. M. (2016). “Chapter six - AMPA receptors as therapeutic targets for neurological disorders” in Advances in protein chemistry and structural biology, ion channels as therapeutic targets, part. ed. Donev R. (Academic Press), 203–261. 10.1016/bs.apcsb.2015.10.004 26920691

[ref48] Lester R. Quarum M. Parker J. Weber E. Jahr C. E. (1989). Interaction of 6-cyano-7-nitroquinoxaline-2,3-dione with the N-methyl-D-aspartate receptor-associated glycine binding site. Mol. Pharmacol. 35, 565–570. 2566902

[ref49] Lewis D. Kenneally M. van denHeuvel C. Byard R. W. (2021). Methamphetamine deaths: changing trends and diagnostic issues. Med. Sci. Law 61, 130–137. doi: 10.1177/0025802420986707 33423599

[ref50] Li Y. Vartanian A. J. White F. J. Xue C.-J. Wolf M. E. (1997). Effects of the AMPA receptor antagonist NBQX on the development and expression of behavioral sensitization to cocaine and amphetamine. Psychopharmacology 134, 266–276. doi: 10.1007/s002130050449 9438676

[ref51] Lynch W. J. Bakhti-Suroosh A. Abel J. M. Davis C. (2021). Shifts in the neurobiological mechanisms motivating cocaine use with the development of an addiction-like phenotype in male rats. Psychopharmacology 238, 811–823. doi: 10.1007/s00213-020-05732-4 33241478 PMC8290931

[ref52] Mead A. N. Stephens D. N. (1998). AMPA-receptors are involved in the expression of amphetamine-induced behavioural sensitisation, but not in the expression of amphetamine-induced conditioned activity in mice. Neuropharmacology 37, 1131–1138. doi: 10.1016/S0028-3908(98)00101-4 9833643

[ref53] Mead A. N. Stephens D. N. (1999). CNQX but not NBQX prevents expression of amphetamine-induced place preference conditioning: a role for the Glycine site of the NMDA receptor, but not AMPA receptors. J. Pharmacol. Exp. Ther. 290, 9–15. 10381753

[ref54] Miyatake M. Narita M. Shibasaki M. Nakamura A. Suzuki T. (2005). Glutamatergic neurotransmission and protein kinase C play a role in neuron–glia communication during the development of methamphetamine-induced psychological dependence. Eur. J. Neurosci. 22, 1476–1488. doi: 10.1111/j.1460-9568.2005.04325.x 16190901

[ref55] Moretti J. Poh E. Z. Rodger J. (2020). rTMS-induced changes in glutamatergic and dopaminergic systems: Relevance to cocaine and methamphetamine use disorders. Front. Neurosci.:14:137. doi: 10.3389/fnins.2020.00137 32210744 PMC7068681

[ref56] Murray C. H. Loweth J. A. Milovanovic M. Stefanik M. T. Caccamise A. J. Dolubizno H.. (2019). AMPA receptor and metabotropic glutamate receptor 1 adaptations in the nucleus accumbens core during incubation of methamphetamine craving. Neuropsychopharmacology 44, 1534–1541. doi: 10.1038/s41386-019-0425-5 31146278 PMC6785134

[ref57] Park W.-K. Bari A. A. Jey A. R. Anderson S. M. Spealman R. D. Rowlett J. K.. (2002). Cocaine administered into the medial prefrontal cortex reinstates cocaine-seeking behavior by increasing AMPA receptor-mediated glutamate transmission in the nucleus Accumbens. J. Neurosci. 22, 2916–2925. doi: 10.1523/JNEUROSCI.22-07-02916.2002 11923456 PMC6758324

[ref58] Picciotto M. R. Kenny P. J. (2021). Mechanisms of nicotine addiction. Cold Spring Harb. Perspect. Med. 11:a039610. doi: 10.1101/cshperspect.a039610 32341069 PMC8091956

[ref59] Polosa R. Benowitz N. L. (2011). Treatment of nicotine addiction: present therapeutic options and pipeline developments. Trends Pharmacol. Sci. 32, 281–289. doi: 10.1016/j.tips.2010.12.008 21256603 PMC5564372

[ref60] Prochaska J. J. Benowitz N. L. (2019). Current advances in research in treatment and recovery: nicotine addiction. Sci. Adv. 5:eaay9763. doi: 10.1126/sciadv.aay9763 31663029 PMC6795520

[ref61] Pushparaj A. Kim A. S. Musiol M. Trigo J. M. Le Foll B. (2015). Involvement of the rostral agranular insular cortex in nicotine self-administration in rats. Behav. Brain Res. 290, 77–83. doi: 10.1016/j.bbr.2015.04.039 25934486

[ref62] Ruda-Kucerova J. Amchova P. Siska F. Tizabi Y. (2021). NBQX attenuates relapse of nicotine seeking but not nicotine and methamphetamine self-administration in rats. World J. Biol. Psychiatry 22, 1–23. doi: 10.1080/15622975.2021.1907714 33787469

[ref63] Ruda-Kucerova J. Amchova P. Babinska Z. Dusek L. Micale V. Sulcova A. (2015a). Sex differences in the reinstatement of methamphetamine seeking after forced abstinence in Sprague-Dawley rats. Front. Psych. 6:91. doi: 10.3389/fpsyt.2015.00091 PMC4492081 26217239

[ref64] Ruda-Kucerova J. Amchova P. Havlickova T. Jerabek P. Babinska Z. Kacer P.. (2015b). Reward related neurotransmitter changes in a model of depression: an in vivo microdialysis study. World J. Biol. Psychiatry 16, 521–535. doi: 10.3109/15622975.2015.1077991 26444572

[ref65] Ruda-Kucerova J. Babinska Z. Amchova P. Stark T. Drago F. Sulcova A.. (2017). Reactivity to addictive drugs in the methylazoxymethanol (MAM) model of schizophrenia in male and female rats. World J. Biol. Psychiatry 18, 129–142. doi: 10.1080/15622975.2016.1190032 27223864

[ref66] Scherma M. Panlilio L. V. Fadda P. Fattore L. Gamaleddin I. Foll B. L.. (2008). Inhibition of anandamide hydrolysis by Cyclohexyl Carbamic acid 3′-Carbamoyl-3-yl Ester (URB597) reverses abuse-related behavioral and neurochemical effects of nicotine in rats. J. Pharmacol. Exp. Ther. 327, 482–490. doi: 10.1124/jpet.108.142224 18725543 PMC2663803

[ref67] Scheyer A. F. Loweth J. A. Christian D. T. Uejima J. Rabei R. Le T.. (2016). AMPA receptor plasticity in Accumbens Core contributes to incubation of methamphetamine craving. Biol. Psychiatry 80, 661–670. doi: 10.1016/j.biopsych.2016.04.003 27264310 PMC5050076

[ref68] Scofield M. D. Heinsbroek J. A. Gipson C. D. Kupchik Y. M. Spencer S. Smith A. C. W.. (2016). The nucleus Accumbens: mechanisms of addiction across drug classes reflect the importance of glutamate homeostasis. Pharmacol. Rev. 68, 816–871. doi: 10.1124/pr.116.012484 27363441 PMC4931870

[ref70] Spuz C. A. Borszcz G. S. (2012). NMDA or non-NMDA receptor antagonism within the Amygdaloid central nucleus suppresses the affective dimension of pain in rats: evidence for hemispheric synergy. J. Pain 13, 328–337. doi: 10.1016/j.jpain.2011.12.007 22424916 PMC3329962

[ref71] Stone T. W. (2021). Relationships and interactions between ionotropic glutamate receptors and nicotinic receptors in the CNS. Neuroscience 468, 321–365. doi: 10.1016/j.neuroscience.2021.06.007 34111447

[ref72] Suto N. Ecke L. E. Wise R. A. (2009). Control of within-binge cocaine-seeking by dopamine and glutamate in the core of nucleus accumbens. Psychopharmacology 205, 431–439. doi: 10.1007/s00213-009-1553-0 19436996 PMC3150710

[ref73] Sutton M. A. Schmidt E. F. Choi K.-H. Schad C. A. Whisler K. Simmons D.. (2003). Extinction-induced upregulation in AMPA receptors reduces cocaine-seeking behaviour. Nature 421, 70–75. doi: 10.1038/nature01249 12511956

[ref74] Tiwari R. K. Sharma V. Pandey R. K. Shukla S. S. (2020). Nicotine addiction: neurobiology and mechanism. J. Pharmacopuncture 23, 1–7. doi: 10.3831/KPI.2020.23.001 32322429 PMC7163392

[ref75] Tobacco Use and Dependence Guideline Panel (2008). Treating tobacco use and dependence: 2008 update Rockville (MD): US Department of Health and Human Services.

[ref9001] Tzschentke T. M. Schmidt W. J. (2003). Glutamatergic mechanisms in addiction. Mol. Psychiatry 8, 373–382. doi: 10.1038/sj.mp.4001269 12740594

[ref76] Ueno F. Suzuki T. Nakajima S. Matsushita S. Mimura M. Miyazaki T.. (2019). Alteration in AMPA receptor subunit expression and receptor binding among patients with addictive disorders: a systematic review of human postmortem studies. Neuropsychopharmacol. Rep. 39, 148–155. doi: 10.1002/npr2.12058 31070872 PMC7292281

[ref77] Unger J. B. Barker J. Cruz T. B. Leventhal A. M. Pentz M. A. (2022). Lucy—novel flavored nicotine gum, lozenges, and pouches: are they misleading consumers? Subst. Use Misuse. 57, 1328–1331. doi: 10.1080/10826084.2022.2076881 35586938 PMC9451008

[ref78] Vanover K. E. (1998). Effects of AMPA receptor antagonists on dopamine-mediated behaviors in mice. Psychopharmacology 136, 123–131. doi: 10.1007/s002130050547 9551768

[ref79] Volkow N. D. Michaelides M. Baler R. (2019). The neuroscience of drug reward and addiction. Physiol. Rev. 99, 2115–2140. doi: 10.1152/physrev.00014.2018 31507244 PMC6890985

[ref9002] Volkow N. D. Wang G. -J. Fowler J. S. Tomasi D. Telang F. (2011). Addiction: Beyond dopamine reward circuitry. Proc. Natl. Acad. Sci. 108, 15037–15042. doi: 10.1073/pnas.1010654108 21402948 PMC3174598

[ref80] Walley S. C. Wilson K. M. Winickoff J. P. Groner J. (2019). A public health crisis: electronic cigarettes, vape, and JUUL. Pediatrics 143:e20182741. doi: 10.1542/peds.2018-2741 31122947

[ref81] Wang F. Chen H. Steketee J. D. Sharp B. M. (2007). Upregulation of ionotropic glutamate receptor subunits within specific mesocorticolimbic regions during chronic nicotine self-administration. Neuropsychopharmacology 32, 103–109. doi: 10.1038/sj.npp.1301033 16452988 PMC2587993

[ref82] Witkin J. M. (1993). Blockade of the locomotor stimulant effects of cocaine and methamphetamine by glutamate antagonists. Life Sci. 53:PL405–PL410. doi: 10.1016/0024-3205(93)90496-P 7902523

[ref83] Wolf M. E. Ferrario C. R. (2010). AMPA receptor plasticity in the nucleus accumbens after repeated exposure to cocaine. Neurosci. Biobehav. Rev. 35, 185–211. doi: 10.1016/j.neubiorev.2010.01.013 20109488 PMC2962767

[ref84] Wooters T. E. Dwoskin L. P. Bardo M. T. (2011). Discriminative stimulus effects of NMDA, AMPA and mGluR5 glutamate receptor ligands in methamphetamine-trained rats. Behav. Pharmacol. 22, 516–524. doi: 10.1097/FBP.0b013e328349aafa 21836462 PMC3354914

[ref85] Xie X. Lasseter H. C. Ramirez D. R. Ponds K. L. Wells A. M. Fuchs R. A. (2012). Subregion-specific role of glutamate receptors in the nucleus accumbens on drug context-induced reinstatement of cocaine-seeking behavior in rats. Addict. Biol. 17, 287–299. doi: 10.1111/j.1369-1600.2011.00325.x 21521425 PMC4384648

[ref86] Yan N. Cao B. Xu J. Hao C. Zhang X. Li Y. (2012). Glutamatergic activation of anterior cingulate cortex mediates the affective component of visceral pain memory in rats. Neurobiol. Learn. Mem. 97, 156–164. doi: 10.1016/j.nlm.2011.11.003 22107830

[ref87] Yasaei R. Saadabadi A. (2022). “Methamphetamine” in StatPearls (Treasure Island (FL): StatPearls Publishing)

[ref88] Zavala A. R. Browning J. R. Dickey E. D. Biswas S. Neisewander J. L. (2008). Region-specific involvement of AMPA/Kainate receptors in Fos protein expression induced by cocaine-conditioned cues. Eur. Neuropsychopharmacol. 18, 600–611. doi: 10.1016/j.euroneuro.2008.04.010 18539009 PMC4798851

